# Extraction of Antioxidant Phenolic Extracts from the Two Main Liquid Effluents of the Olive Industry

**DOI:** 10.3390/foods15193370

**Published:** 2026-09-22

**Authors:** África Fernández Prior, Beatriz Muñíz-Rodríguez, Fátima Rubio Senent, Alejandra Bermúdez-Oria, Juan Fernández-Bolaños, Guillermo Rodríguez-Gutiérrez

**Affiliations:** Instituto de la Grasa, Spanich Scientific Research Council (CSIC), Campus Universitario Pablo de Olavide, Edificio 46, Ctra. de Utrera, km. 1, 41013 Seville, Spain; mafprior@ig.csic.es (Á.F.P.); betriz.mrdz@gmail.com (B.M.-R.); f.r.senent@csic.es (F.R.S.); aleberori@ig.csic.es (A.B.-O.); j.fb.g@csic.es (J.F.-B.)

**Keywords:** antioxidants, hydroxytyrosol, phenolics, biorefinery, bioprocess

## Abstract

The olive industry is experiencing significant growth, which is only held back by the environmental and management issues that its by-products can cause. The two most polluting effluents produced are a new aqueous phase, or ‘alpechín ’, obtained from alperujo, which is the most important waste generated in the olive oil industry, and brine in the table olive industry. The high organic and phytotoxic loads of both, together with the conductivity of the brines, make their reuse difficult and pose a real technological challenge owing to the environmental problems they cause. This study demonstrates that the application of thermo-malaxation (45–65 °C, 60–90 min) followed by three-phase centrifugation (>3500× *g*) allows the removal of the most active phenolics, including hydroxytyrosol, 3,4-dihydroxyphenylglycol, and tyrosol from the alperujo in a 30% solution, yielding a partially detoxified solid phase and a liquid phase rich in the most active phenolics. Furthermore, the application of an industrial chromatographic process (0.6 mm diameter rigid strong-cationic resins) enables the removal of between 50% and 75% of the phenolic load from the liquid fraction of the aforementioned system and from the brines of the table olive industry. The high antioxidant activity of the extracted phenolics was verified using in vitro assays.

## 1. Introduction

Spain is the leading producer of olive oil, accounting for 45% of the global production, and the second largest producer of table olives, accounting for almost 19% of the global production of table olives. At the European level, Spain produces 70% of olive oil and 64% of table olives. According to the Ministry of Agriculture, Fisheries, and Food of Spain (2025) [1]. The main by-products of the olive oil and table olive industries are pruning waste from olive trees, leaves, and olive mill effluents. In Spain, the two-phase extraction system produces alperujo, whereas the table olive industry produces brine and cooking water separately. The olive oil industry also generates effluents, primarily in liquid form, such as washing water, water from vertical centrifuges, and oil decanting water. However, most of these are incorporated into alperujo, thereby increasing its moisture content during production. Alperujo is the semi-solid by-product that consists of approximately 15% pit, 20% pulp and 65% vegetation water [2], with an acidic pH, high salinity, high electrical conductivity [3], and a high content of organic substances such as fats, proteins, sugar, organic acids, cellulose, hemicellulose, pectin, gums, tannins, phenolics and metals such as calcium, potassium, aluminum, iron, magnesium, sodium and strontium [4].

Alperujo is sent directly to the pomace olive oil extractor industry in Spain, which extracts the remaining oil. The dry solid residue obtained is used as biomass for thermal energy production in the company itself or for electricity generation in cogeneration plants that have been incentivized and subsidized by the government [5]. Currently, these subsidies are decreasing, thereby turning dry residues into a latent problem for farmers. The industrial sector is implementing new changes aimed at establishing biorefineries to utilize these by-products. This is the case for the implementation of a novel thermo-malaxation system followed by three-phase centrifugation that is being introduced in olive pomace extractors, with the aim of extracting the remaining oil and separating a liquid fraction to obtain pomace with less moisture and thus reduce energy consumption [6]. This trend is leading to the emergence of a new liquid effluent, the liquid phase enriched in bioactive compounds and sugars, which poses a significant challenge for the olive oil industry owing to the complexity of managing it.

In the table olive industry, brine is produced in greater quantities and presents greater management problems. Brine is the liquid in which table olives are placed for fermentation, and it is composed of sodium chloride in varying amounts depending on the type of preparation. Spent brines, on the other hand, contain a high organic load, including high concentrations of phenolic compounds between 700–1500 mg/L, as well as an acidic pH, conductivity from 70 to 90 mS/cm, and a chemical oxygen demand of 6–21 g O_2_/L [7]. Fermentation brines are responsible for 80–85% of contamination in liquid effluents from the table olive industry, despite representing only 20% of the total volume of wastewater, mainly due to their high salinity. The remaining wastewater volume is primarily generated by the alkali washing process, producing 6 L/kg for Californian types, 3.9 L/kg for Spanish types, and 0.9 L/kg for green types [8]. In Mediterranean countries, the simplest and cheapest way to dispose of brine is through evaporation ponds [9]. Although brine vapors are highly corrosive, some improvements have been made to this system, such as adding turbulence to the water surface, spraying brine, and directing water flows towards the prevailing air to force aeration. Although some companies that handle large volumes of wastewater are resorting to costly systems such as vacuum evaporation, there is currently no widely accepted method for treating effluent water other than using ponds [10].

The main problem with olive by-products is the same: management of high volumes of both brine and alperujo in open-air storage ponds. The core difficulties caused by these evaporation ponds are the need for large areas, generation of unpleasant odors, filtration of compounds into groundwater, impact on surrounding wildlife as they are mistaken for water ponds [4,11], and filtration of nitrogen, ammonium ions, phosphorus, chloride ions, phosphate ions, sulfate ions, potassium, and various metals. Clay soils, which are very common in the Mediterranean basin, retain most of the organic load and nutrients, making them a source of contamination for groundwater in surrounding areas [12]. Despite these drawbacks, pond evaporation systems continue to be used for brines and storage ponds for alperujo, which is subsequently dried for pomace oil extraction. However, the presence of compounds with high nutritional value that could improve the formulation of healthier foods is being overlooked.

Bioactive compounds are natural components with biological activity and, in some cases, high nutritional value [13]. The main bioactive compounds present in olives and their by-products are phenolics, sugars, and triterpene acids. These compounds can have various applications, mainly in the area of food, as preservation methods, functional foods, and animal feed, as well as in the nutraceutical industry in the form of supplements. This is mainly due to the high activity of phenolics and triterpenes, which are anti-inflammatory, immunomodulatory, anti-carcinogenic, antiproliferative, anti-aging, antioxidant, growth stimulants, antiviral, antimicrobial, antibacterial, and antiplatelet agents [14]. Given all this, it is clear why these compounds are of interest to the pharmaceutical and food industries. However, this same antioxidant activity could be an issue in cases where biological treatment is sought because these compounds can be toxic to microorganisms and must be eliminated before fermentation or other bioprocesses are performed [15]. Bioprocesses are essential for achieving comprehensive utilization and maximizing the value of these effluents while ensuring a sustainable, waste-free process. Currently, the most studied compounds are those derived from oleuropein and ligustroside, such as hydroxytyrosol (HT), tyrosol (Ty), and 3,4-dihydroxyphenylglycol (DHPG), whose main characteristic is their hydrophilic nature, which means they are found in greater quantities in olive industry wastewater or in liquid fractions of olive by-products [14]. HT is a compound that has been shown to be a natural antioxidant that can be easily extracted from olive by-products and has numerous benefits, such as chemopreventive cancer treatments, anti-atherogenic activities, skin photoprotection, and significant anti-inflammatory activity [16]. DHPG is a simple phenolic compound with a high antioxidant capacity, which can be even greater than that of HT, which can be unstable in basic media. Other studies have shown that it has a higher antioxidant capacity than ascorbic acid and HT in aqueous media [16]. Finally, Ty is a compound found in smaller quantities in fresh olive pomace, but in similar quantities to DHPG in olive pomace that has accumulated in the ponds [17].

Therefore, it can be concluded that two types of effluents are the most polluting and difficult to manage in the olive-growing industry: brine in the table olive industry and the new liquid fraction from the alperujo in the olive oil industry. The solution is based on the design of a biorefinery, for which it is necessary to apply a bioprocess and undertake a preliminary extraction step to isolate the phytotoxic components, specifically the compounds with high added value [18]. For all these reasons, it is believed that the evolution that is beginning in the sector is moving towards the initial extraction of high added-value components, such as bioactive compounds, followed by the application of bioprocesses or direct use in animal feed. Among the bioprocesses that allow the use of all the resulting materials are anaerobic digestion, biochar formation or other energy sources, and the composting of by-products. In the case of brines, application to biological processes is complicated by salinity, which causes physical and biochemical changes, as well as inhibition generated by phenolics due to their bactericidal effect [7], causing irrigation with water from brines to reduce the yield of trees and crops [10].

This study aimed to improve the management of the two main liquid by-products of the olive industry, brine from the table olive industry and the new liquid phase generated from alperujo from the olive oil industry, which pose a major environmental challenge owing to their volume and organic content. Reducing the phenolic content is important for minimizing phytotoxicity, enabling the use of bioprocesses in olive oil effluent and improving the reuse of brine in the table olive industry. In both cases, this enables the production of phenolic extracts with significant bioactive properties for the formulation of new foods and functional products. In the present study, we analyzed the phenolic content of the two liquid effluents, monitored the three most significant phenolics present, extracted them using an existing industrial system, and investigated the antioxidant capacities of the two phenolic extracts obtained.

## 2. Materials and Methods

### 2.1. Raw Material: By-Products

This study utilized two by-products from the olive industry: brine, alperujo, and the new alpechín (liquid phase), as shown in Figure 1. Olive pomace was processed on a pilot-plant scale using thermo-malaxation at 60 °C for 45 min, followed by three-phase centrifugation to obtain crude pomace oil, an aqueous fraction (or ‘new alpechin’), and a solid fraction. Furthermore, the new alpechin sourced directly from the industry and the brine sourced from the industry were studied as raw materials representing the two most polluting liquid effluents from the olive industry.

#### 2.1.1. Brine

Single-use Manzanilla brine (1200 L) was obtained from the pilot plant of the Instituto de la Grasa, Seville. The brine was stored at 4–6 °C and processed one week after collection. Phenolic compounds were extracted from all samples and were subsequently characterized.

#### 2.1.2. New Alpechin

A total of 300 L of industrial alpechin, derived from Picual olives, was obtained in June 2024 from the experimental olive mill of Instituto de la Grasa (CSIC, Sevilla, Spain). A Pieralisi SPI-7 continuous oil extraction system was used for the extraction. The mill used a three-phase extraction system using a previous thermo-malaxation at 60 °C for 45 min. The samples were stored at 4–6 °C until October 2024, when they were processed. Phenolic compounds were extracted from all the samples and analyzed.

### 2.2. Chemicals

3,4-dihydroxyphenylglycol and 4-β-D-glucoside were purchased from Sigma-Aldrich (Deisenhofer, Germany), Ty from Fluka (Buchs, Switzerland), and HT from Extrasynthese (Lyon Nord, Geney, France). The chemicals used for colorimetric determination, such as trifluoroacetic acid (TFA), anthrone or Folin–Ciocalteu’s phenol reagent, and other compounds such as hydroxymethylfurfural and 2,2-diphenyl-1-picrylhydrazyl, were obtained from Sigma-Aldrich (Madrid, Spain). Sodium bicarbonate (Na_2_CO_3_) and methanol were obtained from Panreac Quimica S.A. (Barcelona, Spain). Acetonitrile was of HPLC grade (Romyl, Teknokroma, Barcelona, Spain).

### 2.3. Phenolic Extraction

#### 2.3.1. Phenolic Extraction of Table Olive Brine

A representative sample of table olive brine was retained, and the remaining volume was processed through chromatographic columns, according to patent WO 2013/007850A1 [19]. In this system, ion exchange resins with a particle size of 0.6 mm are used, maintaining a diameter/height ratio between 2 and 5, at a flow rate of 500 L/h, with a sample/resin ratio of 5 (*v*/*v*), at a working pressure of 0.5 bar, and at ambient temperature. The washing steps to obtain the phenolic extracts were carried out with water, using twice the volume of the bed. One extraction was performed for the new alpechin sample. The process was conducted until the HT content was reduced by 50% relative to the initial concentration, at which point a sample was collected for analysis. Half of the brine was then passed through the column a second time. The phenolic compounds recovered from the columns were concentrated to obtain a phenolic-rich extract, which was subsequently analyzed and compared with the extract obtained from alpechin. After column processing, the brine exhibited an 88% reduction in the HT concentration relative to the initial concentration.

#### 2.3.2. Phenolic Extraction of the New Alpechin

A representative sample of alpechin was retained, and the remaining volume was subjected to extraction [19]. This procedure employs ion-exchange resin chromatographic columns to adsorb and subsequently desorb the phenolic fraction, resulting in the defenolized alpechin. The phenolic compounds recovered from the columns were concentrated to yield a phenol-rich extract, which was analyzed for its composition and antioxidant properties.

### 2.4. Analytical Extractions

Sample preparation for phenolic and sugar determination. The two liquid effluents were centrifuged at 5400× *g* in an Eppendorf™ Minispin™ centrifuge (Hamburg, Germany) at 13,000× *g* for 15 min and subsequently filtered through a 0.45-micron nylon filter. This yielded the sample required for determining the total and individual phenolic and total sugar contents. The extraction of monosaccharide-rich or simple sugars was performed by precipitating the sugars insoluble in the hydroalcoholic mixture, representing the oligosaccharide fraction, as methanol reduces the solubility of polymerized sugars, forming oligo- and polysaccharides. In a microtube, 300 µL of the supernatant obtained from the total sugars was combined with 1200 µL of pure methanol to obtain a methanol: water ratio of 80:20 *v*/*v*. The mixture was vortexed and centrifuged using the aforementioned centrifuge for 5 min. The supernatant containing the simple sugars was collected for further analysis.

### 2.5. Analytical Determinations of Individual Phenolics by HPLC-UV/DAD

The main phenolic compounds in the samples were determined using high-performance liquid chromatography, HPLC-UV/DAD (ultraviolet detector and diode array) equipment (California, USA) and Rheodyne injection valves. An aliquot of 20 μL of the extract samples was injected into the HPLC equipment, using a Teknokroma Mediterranea SEA18 column with an internal diameter of 4.6 mm, a length of 250mm × and a particle size of 5 µm, at room temperature and a flow rate of 1 mL/min. Elution was performed with Milli-Q water (A) with 0.01% trichloroacetic acid and HPLC-grade acetonitrile (B) in the proportions shown in Table 1 and measured at 280 nm. Once the measurement was complete, the data were quantified by integrating the peaks obtained in the chromatogram.

### 2.6. Determination of Total Phenolic Content (TPC)

The total phenolic content was quantified using the Folin–Ciocalteu colorimetric method, following the procedure with minor modifications [6]. The Folin–Ciocalteu reagent reacts with phenolic compounds in an alkaline environment to form a blue complex, the intensity of which is proportional to the concentration of total phenolic compounds. A stock solution of gallic acid (0.25 g/L) in methanol and water (1:1, *v*/*v*) was prepared and used to generate a calibration curve. For sample analysis, 20 µL of each extract was added in quadruplicate to microplate wells, followed by 80 µL of 0.7 M Na_2_CO_3_ and 100 µL of 0.2 M Folin–Ciocalteu reagent. The mixtures were incubated for 15 min at room temperature, and the absorbance was measured at 655 nm using a Bio-Rad iMark microplate reader (Hercules, CA, USA). The results were expressed as gallic acid equivalents (GAE).

### 2.7. Determination of Sugars

The total sugar (TS), monosaccharide (MS), and total oligosaccharide (OS) contents were quantified using the Anthrone method with some modifications [6]. A glucose standard curve (20 mg glucose/100 mL distilled water) was prepared to obtain concentrations ranging from 0.02–0.2 g/L. Aliquots of 100 µL of each sample and their respective dilutions were placed in test tubes in triplicate, along with the glucose standards. To one tube of each set, 200 µL of sulfuric acid was added as a blank, and 200 µL of 0.2% anthrone reagent in sulfuric acid was added to the remaining tubes. The samples were mixed, covered, and incubated in a water bath at 95 °C for 5 min. After cooling, the reaction mixtures were transferred to a microplate, and the absorbance was measured at 630 nm using a Bio-Rad iMark microplate reader (Hercules, CA, USA).

Total sugars were measured directly in aqueous samples, whereas monosaccharide content was determined by direct measurement in the methanol-water extract (8:2, *v*/*v*). The soluble oligosaccharide content was determined by the difference between total sugars and monosaccharides, as oligosaccharides are not soluble in the hydroalcoholic mixture used but are soluble in water.

### 2.8. Determination of pH and Electrical Conductivity

The pH and electrical conductivity of the liquid samples were determined using a pH meter (Inolab, Munich, Germany) equipped with an integrated conductivity sensor. Electrical conductivity was expressed in millisiemens per centimeter (mS/cm).

### 2.9. In Vitro Antioxidant Activity Assays

#### 2.9.1. Antiradical Activity by DPPH radical

2,2-diphenyl-1-picrylhydrazyl (DPPH) radical scavenging activity. The free radical scavenging capacity is a measure of antioxidant activity. The DPPH method was used for each phenolic compound [20]. The results were expressed as EC50 (effective concentration, mg/mL) and calculated for each antioxidant from a calibration curve using linear regression.

#### 2.9.2. Reducing Power

The reducing power was measured for each phenolic extract [21] treated with L^−1^ FeCl_3_ in citric acid. The mixture was measured in quadruplicate using a microplate reader, including a blank without FeCl_3_. The microplate was incubated for 20 min at 50 °C, and after a prewarmed dipyridyl solution in trichloroacetic acid was added, read at 490 nm. The values were expressed as quercetin equivalents (g/L QE) using the following equation determined from linear regression: RP = 0.2172 × A490 − 0.018.

#### 2.9.3. Antiradical Activity by ABTS radical

2,2′-azino-bis(3-ethylbenzothiazoline-6-sulphonic acid) (ABTS): the ABTS assay is based on the scavenging of the ABTS radical (ABTS·+) by antioxidants, compared with the antioxidant potency of Trolox [21]. The results were expressed as the average of the ratios of the slopes of the lines obtained for each sample with Trolox and expressed in terms of Trolox equivalent antioxidant capacity (TEAC) in g/L.

#### 2.9.4. Oxygen Radical Absorbance Capacity (ORAC) Assay

The determination was carried out using the modified ORAC method [22]. Fluorescence was measured using excitation and emission wavelengths of 485 nm and 538 nm, respectively, at 5 min intervals for a total time of 90 min, using a fluorescent plate reader, Fluoroskan Ascent™ FL (Thermo Scientific™, Waltham, MA, USA).

### 2.10. Statistical Analysis

Statgraphics Plus Version 2.1 was used for multivariate comparisons to establish statistical differences between all analyzed parameters. A multivariate analysis of variance (ANOVA) was performed on the data from the different samples, followed by Duncan’s multiple comparison test, using a significance level of *p* < 0.05. All results were obtained in triplicate for colorimetric determinations and in duplicate for chromatography.

## 3. Results and Discussion

The application of the industrial chromatography system resulted, on the one hand, in the production of the same effluent but with a lower phenolic content, known as dephenolized effluent’, which passed through the ion-exchange resin column, where its phenolic and sugar composition was analyzed. In contrast, after eluting the column with water, an aqueous extract rich in phenolics was obtained, concentrated, and its composition and antioxidant activity were determined.

### 3.1. Phenolic Extraction of the New Alpechin

Based on the data presented in Figure 2, it can be observed that after the alpechin or liquid fraction passed through the columns, the pH decreased slightly, likely due to the mildly acidic nature of the column material. There was a significant reduction in soluble solids of 37.2% and in total phenolic content of 64.3%, which is the aim of this system. At an industrial level, a second and even a third pass through the chromatographic column can be carried out, and in each pass, at least half of the phenolics present are removed, meaning that the content can be reduced by up to 75% in two passes. The reductions in HT, DHPG, and Ty were 81.7%, 63.4% and 70%, respectively. On the other hand, sugar levels remained unchanged, which is an advantage, as was already the case with alperujo when applying the thermo-malaxation treatment plus the three-phase process, since sugars promote and enhance the application of a bioprocess, which is one of the objectives of defenolization.

Table 2 shows the moisture content of each sample and the concentrations of the main phenolic compounds present, as well as the concentrations of soluble monosaccharides and oligosaccharides. The significant reduction in moisture content was due to the selective retention of phenolic compounds in the column. A drastic decrease in the concentrations of total and individual phenolics was also observed, and these concentrations could be further reduced if a second pass through the chromatographic system was performed, as previously reported. At the industrial level, only a single pass is currently performed, as this method extracts the greatest quantity of phenolics, allowing extracts to be obtained at a low production cost. With regard to sugars, although their total content does not vary, there is a significant increase in monosaccharides, which may be caused by the acidic nature of the resin used, favoring the hydrolysis of soluble oligosaccharides.

The total phenolic content was lower than 40 g/L reported by other authors, in which the storage time was higher [23]. However, the concentration of DHPG was five times higher than that reported by the authors. In the case of HT and Ty, the concentrations in this study were two to three times lower than those found in a sample that had been stored for two months longer. A similar result was observed for the total sugar concentration, which was six times lower than that reported in the study cited above. Thus, most parameters yielded lower values except for DHPG. The loss of the three main phenolics was significant following the chromatographic process, with 60% of DHPG, 81% of HT and 68% of Ty being removed from the alpechin.

### 3.2. Phenolic Extract from Brine

The extraction process of brines obtained in the table olive industry after the fermentation process (Figure 3) shows that the use of chromatographic columns reduced the conductivity of the effluent by up to 16%, while maintaining a nearly constant and slightly acidic pH. However, the total phenolic content decreased by 38% when passing through the first column and by an additional 35% after the second, corresponding to an overall removal of 60% of the initial phenolic content.

All individual phenolic compounds were reduced after chromatographic extraction, such as DHPG, of which 23% was removed in the first column and 15% of the remaining fraction in the second column, resulting in a total reduction of 35%. For HT, 50% was eliminated in the first column and 75% of the remaining compound was eliminated in the second column, leading to a final reduction of 88%. Ty was decreased by 54% after the first column and 65% after the second, corresponding to an overall 84% removal relative to the initial concentration.

Regarding total soluble sugars, a 10% decrease was observed after the first column and a further 22% after the second, representing a cumulative reduction of 30% in the final product. Monosaccharides were only slightly affected, whereas oligosaccharides decreased by 11% initially and by 27% thereafter, resulting in an overall loss of 35%. This effect may be attributed to sugar retention in the column matrix. Thus, the column system proved effective for phenolic concentration, particularly for Ty and HT.

Table 3 shows the phenolic and sugar concentrations of the samples. The moisture content increased as the dissolved components were removed. With regard to total and individual phenolics, as stated, a significant decrease in all of them is clearly evident. The difference between total phenolics and the main individual phenolics indicates the presence of phenolic polymers and bonds between phenolics and other components, primarily sugars and proteins. In the case of sugars, a small decrease was also observed, which was not as drastic as that for phenolics, but still significant, with oligosaccharides undergoing hydrolysis, while the balance between release and degradation remained stable in the case of monosaccharides.

Although the conductivity of the brine reported by Ferrer-Polonio et al. [7] and Mohamadi et al. [24] was 88 mS/cm and 91–102 mS/cm, respectively, the value obtained in this study (157.14 mS/cm) may differ due to the intrinsic composition of the fermentation brine. The total phenolic content was similar to the reported range of 5.2–15.7 g/L [24]. The HT concentration was in the range reported by other authors, 0.3–3.9 mg/L [7,24]. Ty concentration was lower than the 0.89–1.54 g/L [7] or 1.37–1.52 g/L [24] reported by the same authors. Regarding the total sugar content, the determined concentration slightly exceeded the values of 9.28–9.86 g/L reported by Mohamadi et al. [24].

### 3.3. Antioxidant Activity of Phenolic Extracts

The antioxidant activity of the phenolic extracts obtained from both liquid by-products, the liquid fraction of processed alperujo and the brine, was analyzed. It is important to mention that no sugars were detected using the colorimetric methods employed in either of the two extracts. The two extracts were obtained using a patented chromatographic method currently used in the industry. The analysis shown in Table 4 indicates that the total phenolic compound concentration in the brine was approximately twice that of the olive mill wastewater, although the HT levels were nearly identical.

Figure 4 shows the antioxidant activity results obtained using the four methods. In all four samples, the antioxidant activity of the liquid fraction extract was higher than that of the corresponding brine. At first glance, one might expect the brine extract, with its higher total phenolic compound content, to show greater antioxidant activity; however, this was not observed in the present study. This finding suggests that antioxidant activity may correlate more closely with HT concentration, which was slightly higher in the liquid extract. It should be noted that studies, such as that by Fernández-Prior et al. [16], have reported that HT exhibits strong antioxidant potential. DHPG possesses an even greater antioxidant capacity and may act synergistically with HT to enhance overall antioxidant performance.

The antioxidant activity data indicate that the extracts obtained have high potential, with HT being the main component. Compared with other extracts of the same nature, including olive oil by-products and table olives, twice the scavenging activity was observed for the three free radicals studied, and twice the reducing power of these extracts [16]. The properties of these phenolics extend beyond their antioxidant properties. They also exhibit anti-inflammatory, antiproliferative, antimicrobial, and antiplatelet aggregation activities. Furthermore, natural HT extracts obtained using physical methods exhibit greater antioxidant activity than those obtained by synthesis [16]. This demonstrates that the use of industrial technologies enables the production of extracts with high antioxidant activity for use in the food, cosmetics, and nutraceutical industries, among others, which will add value to the system by not only allowing for the detoxification of by-products but also providing an additional benefit to the value chain. In addition, several studies have demonstrated that once the concentration of phenols in this type of effluent is reduced, bioprocesses such as composting or anaerobic digestion can be applied, thereby enabling the effluent to be fully utilized within an integrated biorefinery.

## 4. Conclusions

The clear trend in the olive oil industry is leading to the production of a worrying liquid effluent, the new alpechin, which, together with brine in the table olive industry, poses a real challenge to improving sustainability in the oil sector. In this regard, the extraction of phenolics represents the first step in the biorefining of these effluents, adding value while reducing their toxicity for subsequent bioprocess applications.

The use of an industrial extraction system based on a physical chromatographic process was able to extract 50% of the phenolics, or even 75% if used twice. This system can also be applied to brine water to remove the same quantity of phenolics. It has also been demonstrated that these effluents are ideal sources for obtaining antioxidant phenolic extracts for use in the food, cosmetics and nutraceutical industries, thereby improving the formulation of new functional foods and enabling the replacement of synthetic antioxidants. In addition, the phenolic content is sufficiently reduced to allow biological processes, such as aerobic or anaerobic fermentation for energy production or composting for the production of agricultural substrates, to be applied with greater confidence in the future.

## Figures and Tables

**Figure 1:**
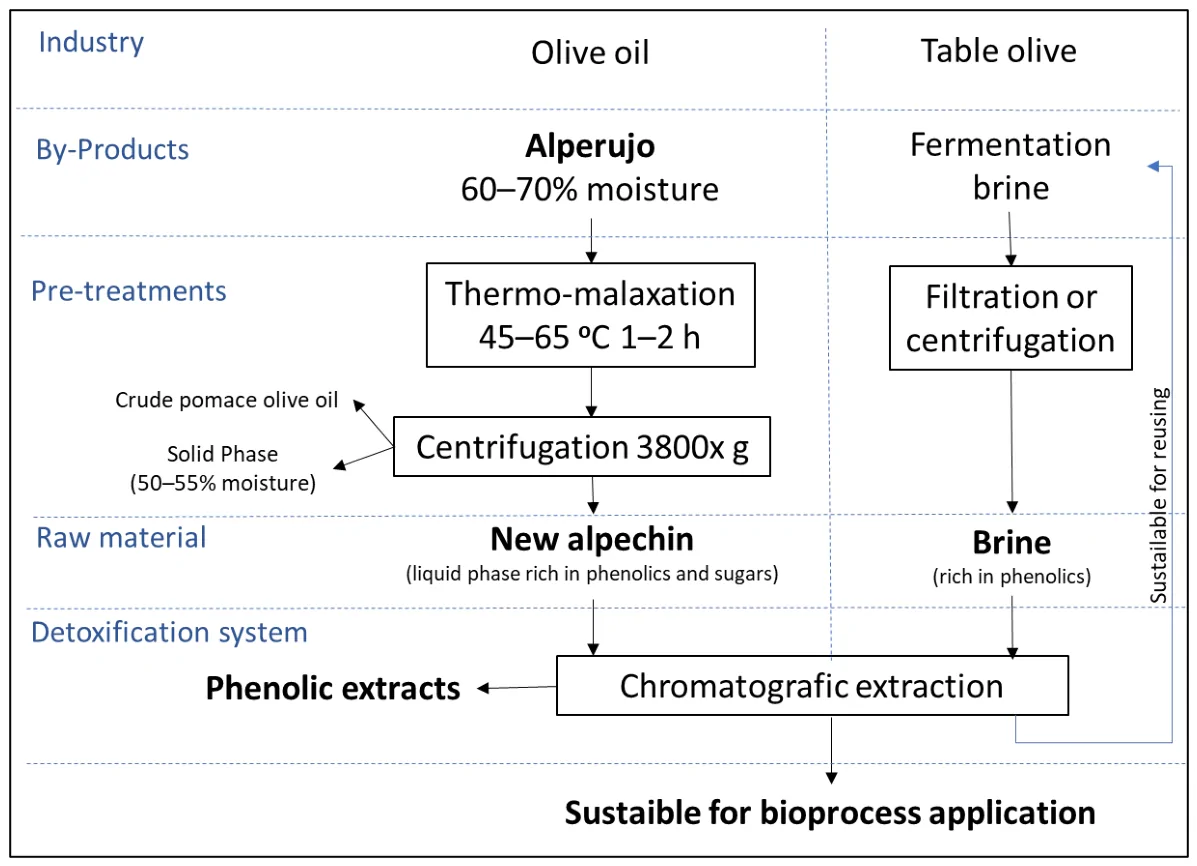
Diagram of the products and processes used in this study.

**Figure 2:**
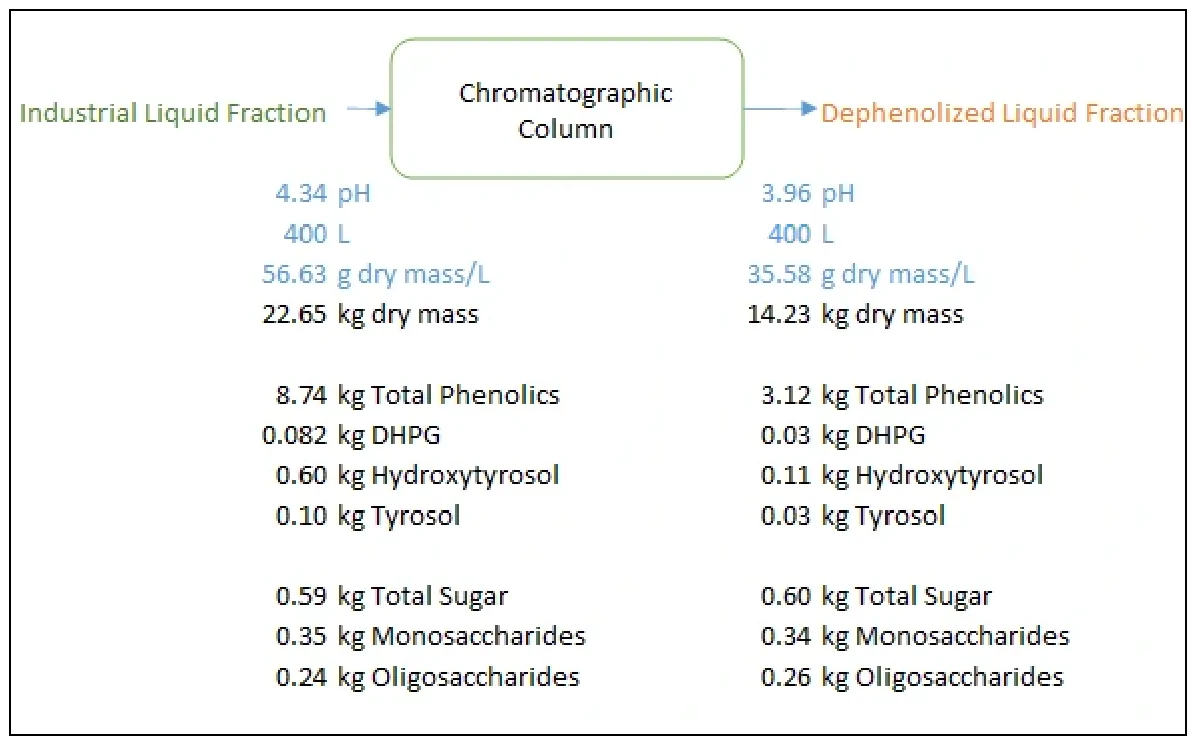
Mass balance, phenolic, and sugar composition of the phenolic extraction by the chromatographic system of the liquid phase or new alpechin.

**Figure 3:**
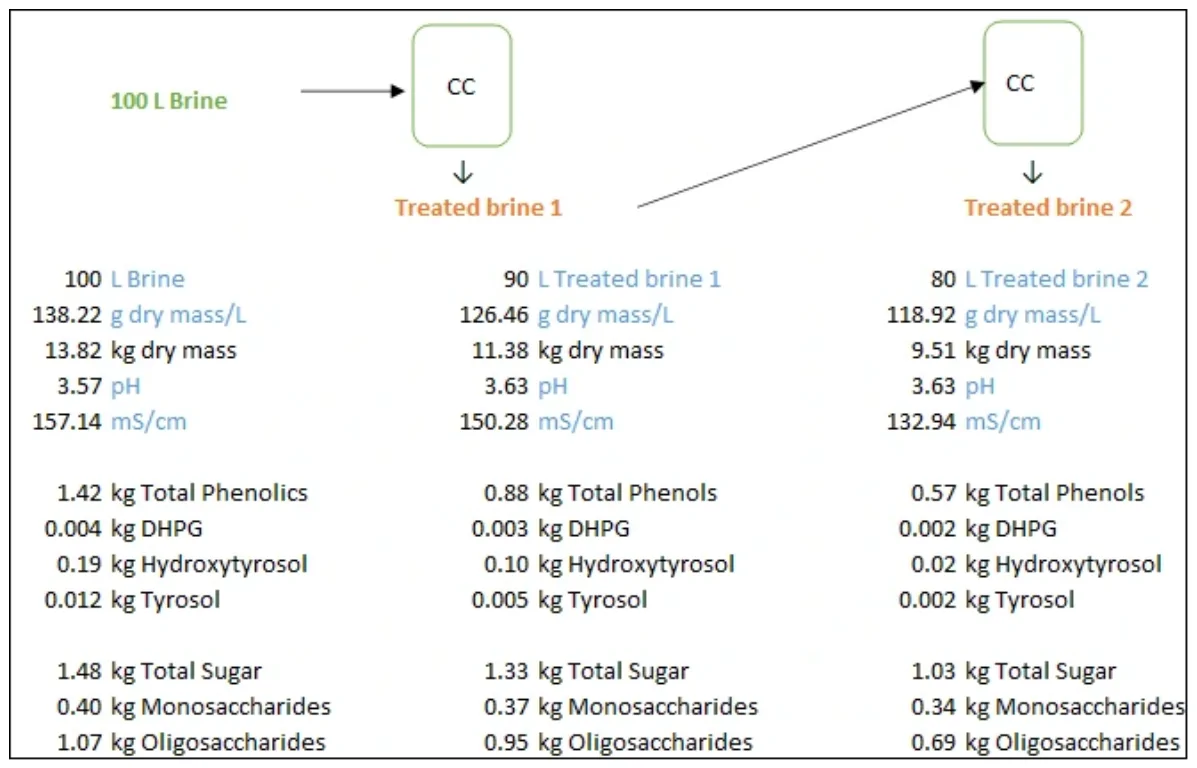
Mass balance, phenolic, and sugar composition of the phenolic extraction by chromatographic system of the brine of olive table industry.

**Figure 4:**
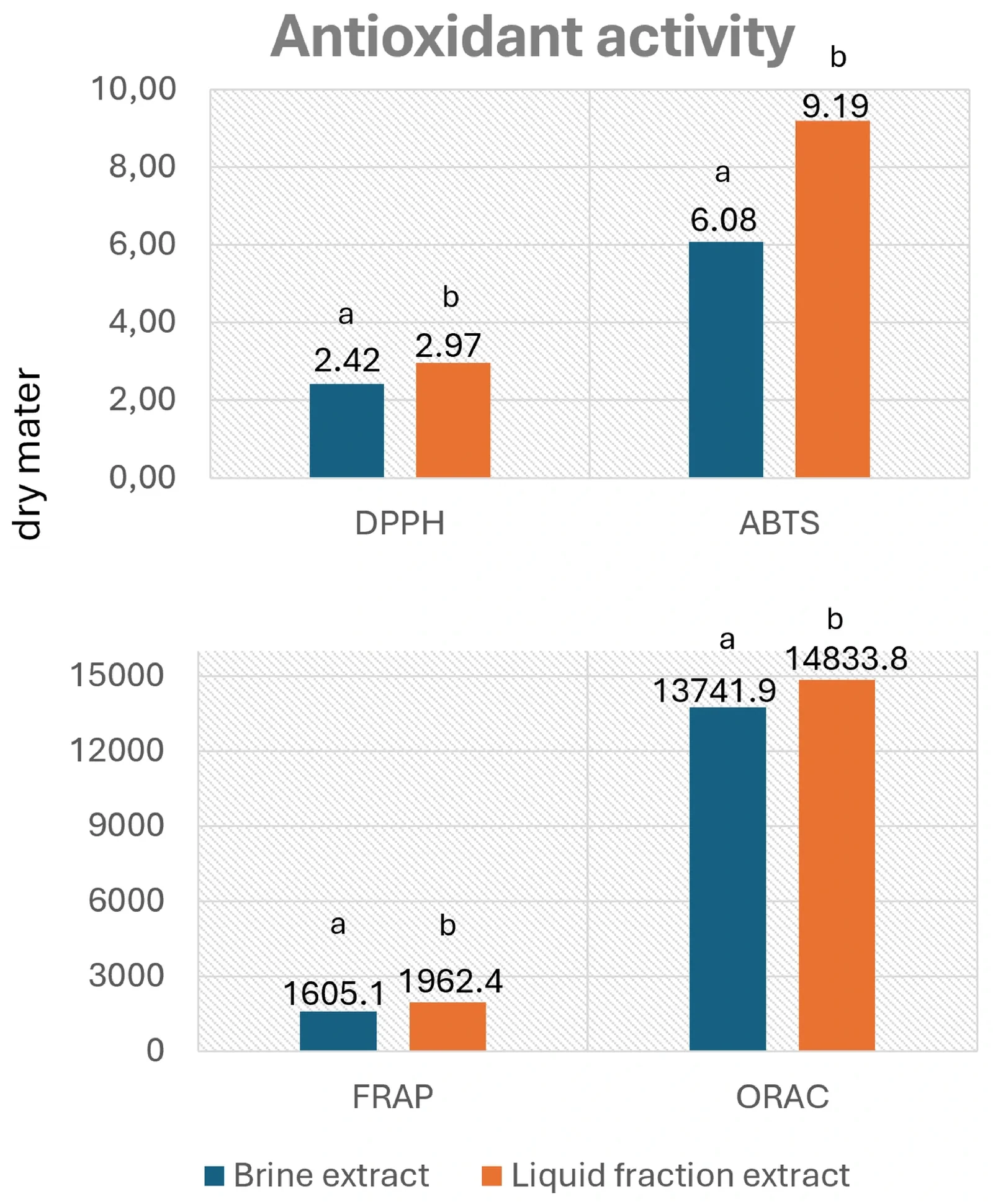
Antioxidant activity of the two phenolic extracts obtained chromatographically from table olive brine and from the liquid fraction obtained from olive pomace via thermo-malaxation and three-phase centrifugation. Three free radical scavenging activities (DPPH, ABTS, and ORAC) and reducing power, as measured by FRAP, were evaluated. Means with the same letter in the same row are not significantly different (*p* < 0.05).

**Table 1:** The gradient was used to measure the phenolic compounds by HPLC.

Time (min)	0	30	45	47	50	55
%A	95	75	50	0	75	95
%B	5	25	50	100	25	5

**Table 2:** Percentage of moisture, concentration of total phenolics (TP), 3,4-dihydroxyphenylglycol (DHPG), hydroxytyrosol (HT), tyrosol (Ty), total sugar (TS), and monosaccharides (MS) in the liquid fraction or alpechin obtained by thermo-malaxation and a three-phase centrifugation system.

	Alpechin (g/L)	Dephenolized Alpechin (g/L)
% Moisture	94.34 ± 1.02 a *	96.44 ± 0.83 b
TP	21.90 ± 1.64 b	7.79 ± 0.60 a
DHPG	0.21 ± 0.01 b	0.08 ± 0.01 a
HT	1.51 ± 0.08 b	0.28 ± 0.01 a
Ty	0.25 ± 0.03 b	0.08 ± 0.01 a
TS	1.48 ± 0.07 a	1.52 ± 0.05 a
MS	0.87 ± 0.05 a	1.21 ± 0.07 b
OS	0.60 ± 0.01 a	0.65 ± 0.02 a

* Means with the same letter in the same row are not significantly different (*p* < 0.05).

**Table 3:** Percentage of moisture, concentration of total phenolics (TP), 3,4-dihydroxyphenylglycol (DHPG), hydroxytyrosol (HT), tyrosol (Ty), total sugar (TS), and monosaccharides (MS) in the brine treated in the Chromatographic Column, all in g/L.

	Brine	Treated Brine 1	Treated Brine 2
% Moisture	86.20 ± 0.32 a *	87.35 ± 0.72 ab	88.11 ± 0.14 b
TP	14.21 ±1.08 c	8.80 ± 0.48 b	5.72 ± 0.92 a
DHPG	0.04 ± 0.00 b	0.03 ± 0.00 ab	0.02 ± 0.00 a
HT	1.90 ± 0.02 c	0.96 ± 0.00 b	0.23 ± 0.01 a
Ty	0.12 ± 0.00 c	0.05 ± 0.00 b	0.02 ± 0.00 a
TS	14.75 ± 0.55 c	13.27 ± 1.09 b	10.34 ± 0.89 a
MS	4.04 ± 0.97 a	3.74 ± 0.58 a	3.42 ± 0.70 a
OO	10.71 ± 1.52 b	9.53 ± 1.67 b	6.92 ± 1.59 a

* Means with the same letter in the same row are not significantly different (*p* < 0.05).

**Table 4:** Total phenolic content and individual phenolic content (g/L) of the two phenolic extracts obtained from the two liquid effluents studied: brine from the table olive industry and the liquid fraction obtained from olive pomace treated in the olive oil industry.

	Brine Extract	Alpechin Extract
Total phenolics (g equivalent Gallic acid/L)	40.46 ± 6.07 b *	20.09 ± 1.21 a
Hydroxytyrosol	16.01 ± 0.54 a	16.3 ± 0.61 a
3,4-dihydroxyphenylglycol	1.2 ± 0.1	2.1 ± 0.1
Tyrosol	1.0 ± 0.0	1.7 ± 0.1

* Means with the same letter in the same row are not significantly different (*p* < 0.05).

## Data Availability

The original contributions presented in this study are included in the article. Further inquiries can be directed to the corresponding author.

## Abbreviations

The following abbreviations are used in this manuscript.

TP	Total phenolics
DHPG	3,4-dihydroxyphenylglycol
HT	Hydroxytyrosol
Ty	Tyrosol
TS	Total sugar
MS	Monosaccharides
OS	Oligossacharides
DPPH	2,2-diphenyl-1-picrylhydrazyl
ABTS	2,2′-azino-bis(3-ethylbenzothiazoline-6-sulphonic acid)
FRAP	Reducing power
ORAC	Oxygen radical absorbance capacity
